# Smokers making a quit attempt using e-cigarettes with or without nicotine or prescription nicotine replacement therapy: Impact on cardiovascular function (ISME-NRT) - a study protocol

**DOI:** 10.1186/s12889-017-4206-y

**Published:** 2017-04-04

**Authors:** Markos Klonizakis, Helen Crank, Anil Gumber, Leonie S. Brose

**Affiliations:** 1 Centre for Sport and Exercise Science, Collegiate Hall, Collegiate Crescent, Sheffield Hallam University, Sheffield, S10 2BP UK; 2 Centre for Health and Social Care Research, Sheffield Hallam University, Sheffield, UK; 3 Institute of Psychiatry, Psychology and Neuroscience, King’s College London, London, UK

**Keywords:** E-cigarettes, Nicotine Replacement Therapy, Microcirculation, Macrocirculation, LDF, FMD

## Abstract

**Background:**

The estimated number of cigarette smokers in the world is 1.3 billion, expected to rise to 1.7 billion by 2025, with 10 million smokers living in the U.K. Smoking is the leading, preventable death-cause worldwide, being responsible for almost 650,000 deaths in the E.U. annually. A combination of pharmacological interventions, including nicotine replacement therapy, bupropion and varenicline, and behavioural support is the most effective approach to smoking cessation. However, even the best methods have high relapse rates of approximately 75% within 6 months. Electronic (or “e-“) cigarettes use battery power to disperse a solution that usually contains propylene glycol or glycerine, water, flavouring and nicotine. E-cigarettes have become the most popular smoking cessation aid in England, however, information on their effects on cardiovascular function is limited and contradictory. As e-cigarettes are not solely nicotine-based products, existing research exploring the effects of nicotine on the cardio-vasculature provides only limited information, while their extensive uptake urges the need of evidence to inform the general public, smokers and policy-makers.

**Methods:**

This is a pragmatic, 3-group, randomised, assessor-blinded, single-centre trial exploring the cardiovascular physiological effects of the use of e-cigarettes (nicotine-free and nicotine-inclusive, assessed separately) combined with behavioural support as a smoking cessation method in comparison to the combination of NRT and behavioural support. The primary outcome will be macro-vascular function, determined by a Flow Mediated Dilatation ultrasound assessment, 6 months following participants’ “quit date”.

**Discussion:**

Participants will be assessed at baseline, 3 days following their self-determined “quit date”, at intervention end (3 months) and 6 months following their “quite date”. Findings are expected to give an indication of the cardiovascular effects of e-cigarettes both in the short- and in the medium-term period, informing the general public, policy holders and researchers, helping to define the future role of e-cigarettes as a smoking cessation aid.

**Trial registration:**

Clinicaltrials.gov NCT03061253. Registered 17th February 2017.

## Background

The estimated number of cigarette smokers in the world is 1.3 billion, expected to rise to 1.7 billion by 2025, with 10 million smokers living in the U.K [1]. Smoking is the leading, preventable death-cause worldwide [2], being responsible for almost 650,000 deaths in the E.U. annually [3].

**Fig. 1:**
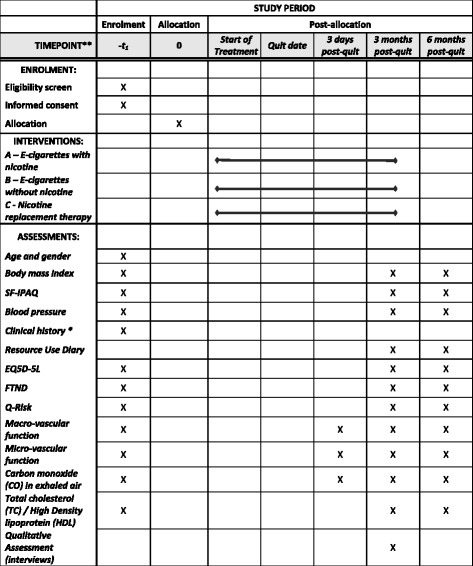
Participant study schedule

Smoking cessation could reduce total cardiovascular disease (CVD) mortality by 36% [2]. This is because the vascular endothelium plays a major role in cardiovascular health and disease (e.g. systemic and pulmonary hypertension, coronary heart disease and stroke; [4], and cigarette smoking has been associated with endothelial dysfunction in otherwise healthy adults [5] – with its detrimental effects ranging from early functional endothelial alterations to late clinico-pathological manifestations of atherosclerotic plaques [2]. The negative effects upon cardiovascular function are not limited to smokers; passive smoking increases heart disease death-risk by almost 30% [6].

A combination of pharmacological interventions, including nicotine replacement therapy (NRT), bupropion and varenicline, and behavioural support is the most effective approach to smoking cessation [7, 8]. However, even the best methods have high relapse rates of approximately 75% within 6 months [7] and although they are offered as part of the UK National Health Service (NHS), these interventions have been reaching fewer smokers (down about 50% from 2010/11 to 2015/16; [9]).

Cigarette smoke contains more than 9000 chemicals; the vast majority of which are the products of tobacco combustion [10]. The most important ones in regards to CVD risk are: oxidizing chemicals, carbon monoxide, volatile organic compounds, particulates, heavy metals and nicotine [11]. Therefore, and taking into consideration that nicotine is highly addictive [11], it is important, where complete abstinence is not possible, to encourage substitution of cigarettes with “cleaner” nicotine-based products to reduce CVD risk and reverse some of the smoking-induced physiological damage [12].

Electronic (or “e-“) cigarettes or vaping devices uses battery power to disperse a solution that usually contains propylene glycol or glycerine, water, flavouring and nicotine [13]. They have become the most popular smoking cessation aid in England [14], while evidence suggests that they are efficacious as smoking cessation aids [15]. However, data on their effects on cardiovascular function is limited i.e. [16] and there is a lack of consensus within the smoking cessation community as to their potential impact: consequently, further research has been requested by the European Parliament [17], the British Medical Association [18], regulatory agencies, clinicians and researchers Lancet; [19], BMC Public Health; [20]. As e-cigarettes are not solely nicotine-based products, existing research exploring the effects of nicotine on the cardio-vasculature (e.g. coronary and peripheral vasoconstriction, intravascular inflammation and deregulation of cardiac autonomic function as well as inhibition of microcirculation; [21]) provides only limited information. Evidence is needed to inform the general public, smokers and policy-makers.

This study aims to assess effects on cardiovascular physiology in smokers making a quit attempt using e-cigarettes (either nicotine-free or nicotine-inclusive) in comparison with smokers making a quit attempt with prescription NRT.

The specific objectives of the study include:

1. The comparison of the effects that nicotine-inclusive e-cigarettes (Group A), nicotine-free e-cigarettes (Group B) and NRT (Group C) have on cardiovascular physiology both acutely (e.g. within 3 days of “quit date”) and in the medium term (6 months following “quit date”), by measuring: a) endothelial-dependent, macro-vascular function and b) endothelial-dependent, micro-vascular function. The inclusion of Group B (nicotine-free e-cigarettes), will allow us to determine whether the e-cigarette vapour constituents have a distinguishable effect on cardio-vasculature against a nicotine-inclusive e-cigarette (Group A).
2. Comparison of the effect of the different treatments on cardiovascular disease risk.
3. An in-depth exploration of the participants’ study experience in a sub-sample of participants.
4. Defining facilitators and barriers of smoking cessation in each of the 3 groups.
5. Assessing the economic cost implications that the approaches have on the NHS and the study participants, by undertaking a Health Economics’ Cost Utility Analysis.

## Methods

### Design

A pragmatic, 3-group, randomised, assessor-blinded, single-centre trial exploring the cardiovascular physiological effects of the use of e-cigarettes (nicotine-free and nicotine-inclusive, assessed separately) combined with behavioural support as a smoking cessation method in comparison to the combination of NRT and behavioural support.

### Study setting

All assessments will be undertaken at the Centre for Sport and Exercise Science (CSES) of Sheffield Hallam University. Behaviour support will be primarily provided in Sheffield Hallam University premises in Collegiate Crescent or City campuses to cover the widest possible Sheffield population, with both locations being easily reached from all Sheffield areas. This setting was chosen to ensure appropriate data collection, to assess its acceptance by participants and monitor their physical and emotional wellbeing, as the university has extensive experience of delivering lifestyle interventions to clinical populations (e.g. cancer, heart failure, aortic aneurysm, chronic venous insufficiency and intermittent claudication). Group C will be referred to NHS smoking cessation clinics in Sheffield (*Yorkshire Smokefree Sheffield*), provided by South West Yorkshire Partnership NHS Foundation Trust.

### Eligibility

*Inclusion Criteria*

- Age > 18 years of either sex,
- Smokers (at least 10 cigarettes/ day for the past year),
- Willing (by declaration) to attempt quit smoking by using the NHS services or e-cigarettes.

*Exclusion Criteria*

- Inability to walk,
- Recent (within 6 months) cardiovascular disease event (e.g. stroke, myocardial infarction) or cardiac surgery,
- Insulin-controlled diabetes mellitus or with coexisting skin conditions, leg ulcers, vasculitis or deep venous occlusion (as these may affect their cardiovascular function),
- Pregnancy,
- Requiring major surgery during the course of the study),
- Contraindications /unsuitability for NRT,
- Current daily use of e-cigarettes,
- Currently undertaking a cessation attempt supported by a smoking cessation clinic,
- Unable to give informed consent.

*Withdrawal*s.

Participants will be considered as withdrawn from the trial if they request to leave the trial, if they are lost to follow-up, or if they die before completing the 12-month follow-up.

Participants will be considered as withdrawals from the allocated treatment if they no longer use the electronic cigarette, or if they no longer receive NRT-based, smoking cessation therapy. Withdrawals from the trial and from the allocated treatment will be included in the analysis by intention-to-treat. Standard care for participants who decide to withdraw from the study will not be affected.

### Recruitment and consent processes

Recruitment from the community in the wider Sheffield area will be via: i) low-cost newspaper and post-office advertisement, ii) posters in local pharmacies, libraries, mosques, churches, and clubs, iii) social media or search engine advertisement (Facebook, Google ads) iv) notices in newsletters or participation in outreach events of community organisations (such as Sheffield U3A and AGE UK), iv) a study website and v) out-reach events in local ethnic community centres or places of worship.

Potential participants will be provided with a study information pack by post, via e-mail or in person, containing an invitation letter (including also the contact details of a research team member), a study information sheet and a stamped, addressed envelope with a tear-off slip to declare their interest (or not) to take part in the study. Following receipt of an “expression of interest” slip/phone call/e-mail, a researcher will contact the patient by telephone and their first visit will be arranged. Smokers who express interest and do not respond within 14 days of the research team posting them an information sheet, will be contacted one more time. During Visit 1 written informed consent will be obtained and eligibility confirmed. The participant schedule for the study is shown in the Fig. 1.

### Resource use diary, randomisation and blinding

After completion of baseline assessments (Visit 1), participants will receive the “resource use” diary together with instructions as of how to complete it. The diary will elicit information on utilisation of NHS and private health care services related to smoking cessation or smoking-related diseases and condition. It is proposed to collects three items of information (1) number of visits, (2) travel time and cost and (3) cost to the patient for the following uses: (a) GP, (b) nurse at smoking cessation clinics, (c) Nurse at smoking cessation clinics (d) Psychologist/Counsellor. Similarly number of times and the costs to the patient for undertaking (1) diagnostic tests (X-rays, Blood test and any other tests) and (2) medications (pain killers, sleeping pills, antibiotics, nicotine patches, any other) related to smoking cessation. Further, number of days of hospitalisation related to smoking related diseases will also be collected.

They be randomised remotely (to ensure allocation concealment) into Groups A (using the nicotine-inclusive e-cigarette), B (using the nicotine-free e-cigarette) and C (being referred to NHS clinic), using a computer programme (nQuery Advisor 6.0, Statistical Solutions, Ireland) to generate stratified block-randomization (by gender and “packet-years” (e.g. number of packs (20 cigarettes per pack)/day x number of years smoked), by an independent statistician. Each participant will be allocated a unique trial number that will remain with him/her throughout the study.

Outcome assessors will be blinded to group allocation and participants will be reminded regularly not to share their group allocation with this person. Statistician and health economist will be blinded to group allocation during the analysis process.

### Sample size

For the purposes of sample size estimation the primary outcome will be macro-vascular function, at 6-months post-“quit date” follow-up, as measured by % Flow-mediated dilatation FMD; [22]. For our calculations we used commercial software (G*Power 3.1.7, HHU of Düsseldorf).

Based on the primary outcome and using data comparing the short-term effects of e-cigarettes on macro-vascular function (measured by %FMD) in smokers and non-smokers (S.D. in the smokers’ group prior to e-cigarettes’ use = 3.23%, expected brachial FMD% difference between NRT group and e-cigarette users = 1.89%; 16). Using G*Power 3.1.7 (HHU of Düsseldorf) we calculated that we will need 66 participants in each group to detect a difference in macro-vascular function at 6 months (significance level = 0.05; power = 80%). Accounting for an estimated 25–30% dropout rate in-line with other e-cigarette-based RCTs [15], we aim to recruit 86 participants in each group (258 in total).

### Intervention

All participants will be asked to define a “quit date” (usually within a week of their baseline assessments or their first appointment at the smoking cessation clinic if in Group C, as per standard practice), on which they will be asked to stop smoking completely. This will be noted by the researcher and define follow-up visits.

Groups A and B will receive complimentary e-cigarette equipment and refills (Tornado V5, Joyetech, Shenzhen, China) at allocation stage, together with instructions on the correct usage of e-cigarettes. They will also receive behavioural support for a 3-month period. The nicotine strength of Group A cartridges will be up to 18 mg/ml nicotine strength, Group B will receive nicotine-free liquid.

Group C participants will be referred to NHS smoking cessation clinics and receive NRT in conjunction with behavioural support. Group C will be reimbursed for their prescription charges for the intervention period.

It is impossible for all study participants to receive behavioural support by the same person or at the same venue as e-cigarettes are not part of the local NHS strategy. To ensure that behavioural support is comparable between groups, all groups will receive the same level and type of behavioural support as currently offered as standard at NHS smoking cessation clinics [23], in the form of regular face-to-face or telephone appointments as per relevant guidelines (e.G. *minimum* of 6 support sessions within the 3-month period, offering practical advice and support, as detailed and structured in reference [23]). All advisors will have completed the same behavioural support training and will have previous NHS-based, SC experience. Behavioural support sessions for Group A and B will take place at easily-accessible locations with a choice offered to participants to match the service experience of Group C.

### Follow-up visits

*VISIT 2:* Within 3 days of “quit date”, vascular assessment of micro- and macro-vascular function *(see Outcomes).*

*VISIT 3:* 3 months past quit date, scheduled to coincide with current local NHS practice, all baseline measurements will be repeated, compliance will be measured in the resource use diaries as per NHS behavioural support strategy [23].

*VISIT 4:* 6 months past quit date, baseline measurements and compliance assessment will be repeated.

### Measures

*Baseline (Visit 1)*

- Demographic data, including age and sex.
- Clinical history (including smoking history), previous operations, comorbidities, current medications, height, weight and blood pressure.
- Fägerstrom Test of Cigarette Dependence FTND; [24].
- EQ5D-5 L to support Health Economics’ assessment; [25].
- Q-Risk to assess risk of CVD; [26].
- Short Form (SF)- International Physical Activity Questionnaire (IPAQ) to assess physical activity; [27].
- Macro-vascular function [22],
- Upper-body micro-vascular function [28],
- “Finger prick” test to calculate the total cholesterol (TC) over High Density lipoprotein (HDL) ratio [29].
- Carbon monoxide (CO) breath levels [30].

### Outcome measures

The primary outcome will be macro-vascular function, a well-established independent predictor of long-term adverse cardiovascular events as determined by an FMD assessment [22], 6 months following participants’ “quit date”.

FMD is a non-invasive, nitric oxide-mediated measure, which independently predicts long-term adverse cardiovascular events [12] and has been used in numerous studies exploring smoking-induced physiological effects i.e. [12, 16]. Baseline scanning to assess resting vessel diameter will be recorded over 3 min, following a 10-min resting period. The brachial artery will be imaged at a location 3 to 7 cm above the antecubital crease to create a flow stimulus in the brachial artery. A sphygmomanometric cuff will be placed on the forearm; the cuff will be inflated at least 50 mmHg above systolic pressure to occlude artery inflow for 5 min. Recordings will commence 30s before cuff deflation and continuing for 3 min after. FMD will be expressed as a change in post-stimulus diameter evaluated as a percentage of the baseline diameter.

Secondary outcomes will include:

- Micro- and macro-vascular function at 3 days to define acute treatment effects and 3 months (interventions’ end).

Microvascular assessments using Laser Doppler Fluximetry and Iontophoresis as described in the literature; [28] will be performed at all study visits, in a temperature-controlled room (22–24 °C) in the ventral surface of the experimental (right) arm extended to the side at heart level. These will be used as indicators of the changes occurring in the endothelial –dependent and –independent vasodilatory function. Heart rate (Sports Tester, Polar, Finland) and blood pressure (left arm; Dinamap Dash 2500, GE Healthcare, USA) will be monitored at 5-min intervals throughout the protocol. The two drug delivery electrodes ((PF383; Perimed AB, Jarfalla, Sweden) will be positioned over healthy looking skin, approximately 4 cm apart with one containing 100 μL of 1% acetylcholine (ACh; Miochol-E, Novartis, Stein) and the other 100 μl of 1% sodium nitroprusside (SNP; Nitroprussiat, Rottapharm). A battery-powered iontophoresis controller (PeriIont PF382b; Perimed AB) will be used to provide the charge needed for ACh and SNP delivery. A 4 min stable recording of baseline flux will be followed by administration of the two agents according to the following protocol: 0.2 mA for 10 s (i.e., 2 mC), 0.2 mA for 15 s (i.e., 3 mC), 0.2 mA for 20 s (i.e., 4 mC), and 0.3 mA for 20 s (i.e., 6 mC), happening between 4-min intervals. To obtain an index of skin blood flow, cutaneous red cell flux will be measured by placing an iontophoresis laser-Doppler probe (PF481–1; Perimed AB), connected to a laser Doppler fluxmeter (PF5001; Perimed AB).

b) Micro-vascular function at 6 months (measured as described for a).
c) Smoking status at 3 and 6 months, self-reported and biochemically validated by exhaled air measurement of <10 ppm Carbon monoxide in line with the Russell Standard [30].
d) change in CVD risk at 3 and 6 months compared with baseline, measured using Q-risk
e) Health Economic effects (assessed using EQ5D-L and a resource use diary) at 6 months. The EQ-5D is a generic measure of health status, where health is characterized on five dimensions (mobility, self-care, ability to undertake usual activities, pain, anxiety/depression).The EQ-5D has been validated in the UK and has been used in other clinical trials i.e. [31].
f) Total cholesterol (TC) and High Density lipoprotein (HDL) via finger prick blood sample at 3 and 6 months; HDL is negatively affected by smoking [29], and improvements in the TC/ HDL ratio can be demonstrated 3 weeks after quitting [32].

### Qualitative exploration

- We aim to explore the following topics:
- Factors that help smokers using e-cigarettes to quit smoking and deter smokers using e-cigarettes from quitting smoking, responding directly to a recent Public Health England call for more research on those areas [13].
- Experiences of treatment and advice given.
- Participant’s preference for trial allocation.
- Experiences of study participation
- Participant acceptability of the intervention and study procedures.
- Methodological approach: Semi-structured in-depth interviews conducted either face to face or via telephone at 3-months post-group allocation with twelve to sixteen participants recruited using purposive sampling (mixture of genders, younger and older participants from all three intervention groups).

### Data analysis

SPSS 21.0 will be used to enter quantitative data and cleaning the data and to carry out missing values imputation and statistical analysis. Data will be reported and presented according to the revised CONSORT statement [33].

Analyses will be performed on an intention-to-treat basis. Double data entry will be implemented to to promote data quality. Where applicable, tests will be two-tailed with statistical significance being set at *p* ≤ 0.05. Baseline demographic (i.e. age, gender, HSI), physical measurements (e.g. weight, height, BMI), carbon monoxide and nicotine dependence levels, as well as Q-Risk, SF-IPAQ and EQ5D-L will be assessed for comparability between groups.

An Analysis of Variance (ANOVA) will be used to compare mean macro- and micro-vascular function between groups. A 95% confidence interval (CI) for the mean difference in these parameters between the three groups will also be calculated. In the event of differences between groups with respect to baseline measurements, multiple regression will be used to adjust the treatment effect for these variables, with the stratification covariates being included in the analysis. The ordinary least squares adjusted regression coefficient estimate for the e-cigarette groups’ parameter, along with its 95% CI, will then be reported. Secondary outcomes at 6-months will be analysed similarly. For repeated assessments at baseline, and at all follow-up sessions a summary measure (e.g. “Area under the Curve”) will be calculated for each participant.

Finally, a further subgroup analysis of those who ceased smoking completely will be undertaken and results will be compared amongst the three groups.

For qualitative data analysis, audio recording of the interviews will be made, transcribed verbatim, and the five stages of framework analysis of familiarisation, identifying a thematic framework, indexing, charting, and mapping will be followed [34, 35].

### Further information on health economics’ analysis

We will undertake a comprehensive cost-utility analysis with both NHS and societal perspectives. We will make an assessment and valuation of costs (direct and indirect costs) and benefits (QALY gains) as a result of the 3-month smoking cessation intervention. The major cost drivers will be patient’s use of NHS and private health services, the patient’s time and their travel costs and intervention delivery costs. To achieve this we will calculate:

- Cost of intervention (e.g. staff time, resources and equipment used and room costs).
- Costs to participants (being based on the distance to SHU or smoking cessation clinics and travel costs, time taken-off from work to attend sessions and any additional liquids or NRT-based medication bought by participants).
- Difference in resource use between groups (elicited from the resource use diary).
- Differences in patient experiences and health related outcomes between intervention and control groups (assessed via the EQ5D-5 L questionnaire, which using the standardised general population based tariff, would allow us to have its scores converted into QALYs and then undertake a comparison between study groups).
- Incremental Cost-Effectiveness Ratio (ICER) (derived from relating differences in costs to benefits between study groups during the 6-month period).

We will use standard published sources for unit costs where available and supplement this with local sources if required. We will use imputation methods for missing data, analysis of uncertainty and non-normal distributions: i.e. standard statistical analysis of costs will be performed to test for statistical significance of results, while bootstrapping would be performed to estimate the CI of average interventional costs.

### Safety monitoring

Study investigators will be responsible for recording adverse events and for determining the seriousness, severity, causality and expectedness of any such events.

The study Primary Investigator will monitor patient safety within the trial and will be responsible for reviewing any adverse events occurring as part of the trial in collaboration with a delegated clinician and confirming that they have been appropriately classified.

Participants will be asked to contact the study team to inform them about adverse events if and when they occur. Study investigators will also question participants about the occurrence of adverse events during each participant study visit.

We will record all serious adverse events, as well as all non-serious adverse events that are deemed to be related to participation in the research, during the period between provision of informed consent through to 12 months after randomisation.

Serious adverse events are defined as any untoward medical occurrence that results in one of the following criteria:

- Results in death,
- Is life threatening (i.e. event in which patient is at risk of death at the time of the event occurring),
- Requires unplanned or prolonged hospitalisation (unplanned refers to emergency hospitalisations. Prolonged hospitalisation is deemed to be where a patient’s stay is longer than expected (e.g. patient is operated on as day case but remains in hospital overnight)
- Results in persistent or significant disability or incapacity

A non-serious event in the context of this trial will be any untoward medical occurrence to the participant that is related to the participants’ involvement in the study, but does not fulfil any of the serious adverse event criteria.

### Data handling and record keeping

The study will adhere to Data Protection Act (1998). Data from this study will be anonymised and stored in password protected computer systems accessible only by the members of the research team, in order to guarantee confidentiality to participants. Paper forms will be stored in locked filing cabinets. All these will be kept in a secured and continuously monitored building facilities where the project will be conducted (Collegiate Hall, Collegiate Crescent, Sheffield Hallam University). Study material will remain in this location for data entry and storage. Patient names will not be used to identify any data. Instead, participants will be assigned a study identification number in order to anonymise their information. All data will be anonymised according to the NHS Code of Confidentiality and GMC Good Medical Practice while publications will not contain identifiable personal data. Furthermore, only research team members will have access to participants’ personal data during the project, while data will be analysed locally. Data will be kept for a 7-year period following the study completion to allow the study team to re-analyze it in case of the development of a new method of analysis.

## Discussion

Findings of this study, are expected to give an indication of the cardiovascular effects of e-cigarettes both in the short- and in the medium-term period. Our work will inform the general public, policy holders and researchers, helping to define the future role of e-cigarettes as a smoking cessation aid.

## Abbreviations

ACh: Acetylcholine chloride
AG: Dr. Anil Gumber
ANOVA: Analysis of Variance
BMI: Body Mass Index
CI: Confidence intervals
CO: Carbon monoxide
CSES: Centre for Sport and Exercise Science
CVD: Cardiovascular disease
EQ-5D-5: L EuroQol 5-dimension (5 L) health status questionnaire.
FMD: Flow-mediated dilatation
FTND: Fägerstrom Test of Cigarette Dependence
GP: General practitioner
HC: Dr. Helen Crank
HDL: High Density lipoprotein
ICER: Incremental Cost-Effectiveness Ratio
ISME-NRT: Smokers making a quit attempt using e-cigarettes (vaping devices) or prescription nicotine replacement therapy: Impact on cardiovascular function
LB: Dr. Leonie Brose
MK: Dr. Markos Klonizakis
NHS: National Health Service
NRT: Nicotine replacement therapy
QALY: Quality-adjusted life-year
SF-IPAQ: Short Form- International Physical Activity Questionnaire.
SNP: Sodium nitroprusside
TC: Total cholesterol
